# Nose to brain delivery of antiretroviral drugs in the treatment of neuroAIDS

**DOI:** 10.1186/s43556-020-00019-8

**Published:** 2020-12-10

**Authors:** Anupam Sarma, Malay K. Das

**Affiliations:** 1 Drug Delivery Research Laboratory, Department of Pharmaceutical Sciences, Dibrugarh University, Dibrugarh, Assam 786004 India; 2 Pratiksha Institute of Pharmaceutical Sciences, Guwahati, Assam 781026 India

**Keywords:** Intranasal delivery, neuroAIDS, Targeted delivery, HIV, Nanoparticle, NLCs

## Abstract

NeuroAIDS (Neuro Acquired Immunodeficiency Syndrome) or HIV (Human Immunodeficiency Virus) associated neuronal abnormality is continuing to be a significant health issue among AIDS patients even under the treatment of combined antiretroviral therapy (cART). Injury and damage to neurons of the brain are the prime causes of neuroAIDS, which happens due to the ingress of HIV by direct permeation across the blood-brain barrier (BBB) or else via peripherally infected macrophage into the central nervous system (CNS). The BBB performs as a stringent barricade for the delivery of therapeutics drugs. The intranasal route of drug administration exhibits as a non-invasive technique to bypass the BBB for the delivery of antiretroviral drugs and other active pharmaceutical ingredients inside the brain and CNS. This method is fruitful for the drugs that are unable to invade the BBB to show its action in the CNS and thus erase the demand of systemic delivery and thereby shrink systemic side effects. Drug delivery from the nose to the brain/CNS takes very less time through both olfactory and trigeminal nerves. Intranasal delivery does not require the involvement of any receptor as it occurs by an extracellular route. Nose to brain delivery also involves nasal associated lymphatic tissues (NALT) and deep cervical lymph nodes. However, very little research has been done to explore the utility of nose to brain delivery of antiretroviral drugs in the treatment of neuroAIDS. This review focuses on the potential of nasal route for the effective delivery of antiretroviral nanoformulations directly from nose to the brain.

## Introduction

More than 35 years ago, HIV was reported as a unique infection with apparent death [1–3]. Menace of HIV infection can be gauged with the fact that every week, around 5500, young women aged 15-24 years become infected with HIV. The UNAIDS confirms 75.7 million populations have been infected with HIV till now from the time of this epidemic has first evolved along with the death of 32.7 million for AIDS-related illnesses. Globally 38 million populations were living with HIV in 2019, including 36.2 million adults and 1.8 million children. About 81% of all people living with HIV knew their HIV status, whereas about 7.1 million populations did not realize that they were infected with HIV. At the end of 2019, 25.4 million communities were taking antiretroviral therapy (ART), which is 67% of all people living with HIV accessing treatment, comprising 68% adults and 53% of children. Among this, 73% of female adults aged above 15 years had access to treatment, while only 61% of male adults aged above 15 years had access to treatment. About 85% of HIV infected pregnant women had been treated with ART in 2019. It was also reported that 1.7 million populations were newly infected with HIV, while 690 thousand people died from AIDS-related illnesses in 2019 [4].

Earlier in AIDS patients, the HIV infection was used to treat without giving much attention to neuropsychiatric complications. More emphasis on the disease opportunistic infections and neurological disorders could be detected even if in the first case of HIV/AIDS [5, 6]. Neurological complications have been found in more than 50% of HIV seropositive cases. The central nervous system (CNS) and peripheral nervous system (PNS) or both are affected in these neurological complications. About 80% of autopsies of AIDS patients show neuropathology [7, 8]. The symptoms can be noticed clinically with the development of AIDS. In AIDS patients, CD4+ T-lymphocyte counts are <200 cells/μl of blood and HAD (HIV-associated dementia), HIV wasting syndrome are prevalent [7, 9].

Nearly 15% of the world’s disease comprises of neuropsychiatric disorders [10, 11]. In the last few years, neuropsychiatric disorders have been observed among HIV seropositive as well as AIDS patients. About 30–50% of AIDS patients experiences neuropsychiatric complications collectively called neuroAIDS [12, 13]. In some cases, decreased functionality of brain and movement skills, along with behaviour and mood shifts, has been observed [14]. Collectively these abnormalities are termed as HIV associated Neurocognitive Disorder (HAND). The diagnosis of HAND is done by assessing brain functionality and ststus of neurophysiology. HAND is further classified as asymptomatic HAND including cognitive complications such as asymptomatic neurocognitive impairment (ANI) or minor neurocognitive disorder (MND), and HIV associated dementia (HAD). The occurrence of HAD (2-4 %) has been observed to be decreased with combined antiretroviral therapy (cART). However, the ANI and MND are remains persist (50-80% of the total HAND patients) [15, 16]. ANI comprises of HIV associated cognitive impairment along with at least two from the cognitive domain such as memory power, speed of brain processing, allart and attention, sensory-perceptual, and motor skills, which generaly does not hinder the everyday activity of the patients,. The MND is marked by HIV induced cognitive disorder, with reduced functionality of brain, motility, and behavioural changes [17].

Indisputably, the improvement in anti-HIV medicines has increased the life expectancy among HIV positive peoples. AIDS patients under medication even last for 20 years or more after initial exposure to HIV. The variety of neuropsychiatric disorders among AIDS patients were indicative of advanced stages of HIV infections. Unfortunately, neuroAIDS occurs at a productive age (30–40 years) of patients. Neuropsychiatric disorders take increased health care costs, and these patients become inefficient for any fruitful work [18, 19].

With the advancement of diagnostic techniques for HIV and monitoring of disease progression United States Food and Drug Administration (US FDA) has approved numerous anti-HIV drugs (**Table** **1**) and many are in the clinical trial (**Table** **2**). Despite the availability of several ARTs, neuroAIDS becomes challenging to control due to low bioavailability of the therapeutic agents in the brain. Hence, there is no evidence for a decline in incidences of neuroAIDS globally. Therefore, there is urgency for nanotherapeutics, which may cross the blood-brain barrier (BBB) to release sufficient amounts of a drug in the brain to curb neuroAIDS. Emerging nanotechnology can very well utilize nanoscale particles as virucidal agents. Therefore, active drug targeting systems, including nanoparticles, are an issue of urgent concern [26]. Nose to brain delivery bay is a promising alternative to target antiretroviral drugs into the brain. Improved antiretrovirals (ARVs) have reduced morbidity and mortality among HIV seropositive. An increase in life span possibly leads to neuroAIDS among long-term HIV survivors. Hence the incidence of neuroAIDS cannot be overlooked, as it may spoil the future with its serious consequences [27].

**Table 1:** US FDA approved antiretroviral drugs and status of their nose to brain delivery

Pharmacological Class	Generic Name	US FDA Approval Date	Investigation on Nose to brain delivery
**Nucleoside Reverse Transcriptase Inhibitors (NRTIs)**			
NRTIs prevent reverse transcription of viral RNA into DNA by blocking the reverse transcriptase enzyme	Abacavir (ABC)	1998/12/17	No
	Emtricitabine (FTC)	2003/07/02	No
	Lamivudine (3TC)	1995/11/17	No
	Tenofovir disoproxil fumarate (TDF)	2001/10/26	Yes [20]
	Zidovudine (azidothymidine, AZT, ZDV)	1987/03/19	Yes [21, 22]
**Non-Nucleoside Reverse Transcriptase Inhibitors (NNRTIs)**			
NNRTIs prevent reverse transcription of viral RNA into DNA by blocking the reverse transcriptase enzyme	Doravirine (DOR)	2018/08/30	No
	Efavirenz (EFV)	1998/09/17	Yes [23]
	Etravirine (ETR)	2008/01/18	No
	Nevirapine (NVP)	1996/06/21	No
	Rilpivirine (RPV)	2011/05/20	No
Protease Inhibitors (PIs)			
PIs block HIV protease enzyme responsible for viral maturation and infectivity	Atazanavir (ATV)	2003/06/20	No
	Darunavir (DRV)	2006/06/23	No
	Fosamprenavir (FPV)	2003/10/20	No
	Ritonavir (RTV)	1996/03/01	No
	Saquinavir (SQV)	1995/12/06	Yes [24]
	Tipranavir (TPV)	2005/06/22	No
Fusion Inhibitors			
Fusion inhibitors block the fusion of HIV with CD4 cells of the immune system; thereby prevent its entry into the immune cell	Enfuvirtide (T-20)	2003/03/13	No
CCR5 Antagonists			
CCR5 antagonists block CCR5 coreceptors on the surface of specific immune cells through which HIV enter into the cells	Maraviroc (MVC)	2007/08/06	No
Integrase Inhibitors			
Integrase inhibitors prevent the integration of viral DNA with host DNA by blocking HIV integrase enzyme	Dolutegravir (DTG)	2013/08/13	No
	Raltegravir (RAL)	2007/10/12	No
Attachment Inhibitors			
Attachment inhibitors bind to the gp120 protein on the outer surface of HIV, thereby prevent the internalization of HIV into CD4 cells	Fostemsavir (FTR)	2020/07/02	No
Post-Attachment Inhibitors			
Post-attachment inhibitors block CD4 receptors on the surface of certain immune cells that HIV needs to enter the cells.	ibalizumab-uiyk (Hu5A8, IBA, Ibalizumab, TMB-355, TNX-355)	2018/03/06	No
Pharmacokinetic Enhancers			
Pharmacokinetic enhancers are used in HIV treatment to increase the residence time of other anti-HIV drug of the HIV regimen for better efficacy of an HIV medicine	Cobicistat (COBI, c)	2014/09/24	No
**Combination HIV Medicines**			
Combination HIV medicines contain two or more HIV medicines from one or more drug classes	abacavir and lamivudine	2004/08/02	No
	abacavir, dolutegravir, and lamivudine	2014/08/22	No
	abacavir, lamivudine, and zidovudine	2000/11/14	No
	atazanavir and cobicistat	2015/01/29	No
	bictegravir, emtricitabine, and tenofovir alafenamide	2018/02/07	No
	darunavir and cobicistat	2015/01/29	No
	darunavir, cobicistat, emtricitabine, and tenofovir alafenamide	2018/07/17	No
	dolutegravir and lamivudine	2019/04/08	No
	dolutegravir and rilpivirine	2017/11/21	No
	doravirine, lamivudine, and tenofovir disoproxil fumarate	2018/08/30	No
	efavirenz, emtricitabine, and tenofovir disoproxil fumarate	2006/07/12	No
	efavirenz, lamivudine, and tenofovir disoproxil fumarate	2018/03/22	No
	elvitegravir, cobicistat, emtricitabine, and tenofovir alafenamide	2015/11/05	No
	elvitegravir, cobicistat, emtricitabine, and tenofovir disoproxil fumarate	2012/08/27	No
	emtricitabine, rilpivirine, and tenofovir alafenamide	2016/03/01	No
	emtricitabine, rilpivirine, and tenofovir disoproxil fumarate	2011/08/10	No
	emtricitabine and tenofovir alafenamide	2016/04/04	No
	emtricitabine and tenofovir disoproxil fumarate	2004/08/02	No
	lamivudine and tenofovir disoproxil fumarate	2018/02/28	No
	lamivudine and zidovudine	1997/09/27	No
	lopinavir and ritonavir	2000/09/15	No

**Table 2:** Investigational antiretroviral drugs in clinical trial [25]

Sl. No.	Investigational drug	Intervention	ClinicalTrials.gov Identification Number	Clinical Phase	Sponsor	Status
1	3BNC117 & 10-1074	An Open Label, Randomized Safety and Antiretroviral study of 3BNC117 and 10-1074 in HIV-infected Individuals.	NCT03526848	Phase 1	Rockefeller University	Recruiting
2	Albuvirtide	A Multicenter Study to Establish the Dosage, Safety and Antiviral Activity of Combination Therapy With Albuvirtide and 3BNC117.	NCT03719664	Phase 2	Frontier Biotechnologies Inc.	Recruiting
3	Aldesleukin	An Open Label antiretroviral study of Interleukin-2 in HIV Reservoirs.	NCT03308786	Phase 2	Case Western Reserve University	Completed
4	Cabotegravir	A Double Blind Safety and Efficacy Study of Long-Acting Injectable Cabotegravir for Pre-Exposure Prophylaxis in HIV-Uninfected Women.	NCT03164564	Phase 3	National Institute of Allergy and Infectious Diseases (NIAID)	Recruiting
5	Dapivirine (DPV) Vaginal Ring (VR)	A Randomized, Open Label Safety study of Dapivirine Vaginal Ring and Oral TRUVADA® Use in Pregnancy.	NCT03965923	Phase 3	National Institute of Allergy and Infectious Diseases (NIAID)	Recruiting
6	Elpida®	Multicentre, Open-label, Post-approval Observational Study of Elpida® Used in the First Line Therapy for HIV-1 Infected Patients.	NCT03706950	-	Viriom	Recruiting
7	Islatravir (MK-8591)	A Double-Blind, Placebo-Controlled Study to Evaluate the Safety, Tolerability, and Pharmacokinetics of Oral MK-8591 Once-Monthly in Participants at Low- Risk for HIV-1 Infection	NCT04003103	Phase 2	Merck Sharp & Dohme Corp.	Recruiting
8	Lefitolimod	A Randomized, Placebo-controlled study with TLR9 Agonist and Broadly Neutralizing Antibodies for Reservoir Reduction and Immunological Control of HIV Infection.	NCT03837756	Phase 2	University of Aarhus	Recruiting
9	Leronlimab (PRO 140)	A Multi-center, Open Label study to assess the efficacy, clinical safety and tolerability parameters of PRO 140 in combination with failing ART in Treatment-Experienced HIV-1 Subjects	NCT03902522	Phase 2/ Phase 3	CytoDyn, Inc.	Recruiting
10	Panobinostat	A Pilot Study to Assess the Safety and Efficacy of Combined Administration With PEGylated Interferon-alpha2a and the Histone Deacetylase Inhibitor (HDACi) Panobinostat for Reducing the Residual Reservoir of HIV-1 Infected Cells in cART-Treated HIV-1 Positive Individuals	NCT02471430	Phase 1/ Phase 2	Massachusetts General Hospital	Recruiting
11	Somatotropin	A Study to Assess the Effect of Recombinant Human Growth Hormone on the Size of the Replication-competent Viral Reservoir in HIV-infected Individuals on Suppressive Antiretroviral Therapy	NCT03091374	Phase 2	McGill University Health Centre/Research Institute of the McGill University Health Centre	Recruiting
12	Chidamide	A study to access the safety and effectiveness of the combination of Chidamide with Chimeric Antigen Receptor (CAR)-T or T cell receptor (TCR)-T cell therapy on HIV patients based on cART.	NCT03980691	Phase 1	Guangzhou 8th People's Hospital	Recruiting
13	UB-421	A Randomized, Open-label, Controlled Study, to Evaluate the Safety of UB-421 in Combination With Standard Antiretroviral Therapy (ART) and the Efficacy of HIV Reservoir Reduction as Compared With ART Alone in ART Stabilized HIV-1 Patients	NCT03743376	Phase 2	United BioPharma	Recruiting
14	Vedolizumab	A study to assess the safety of an analytical treatment interruption (ATI), and to determine whether vedolizumab can control HIV infection in the bloodstream without the use of ART.	NCT03147859	Phase 2	Ottawa Hospital Research Institute	Recruiting
15	Vorinostat	A Study to Evaluate the Effects of Vorinostat and HIV-1 Antigen Expanded Specific T Cell Therapy (HXTC) on Persistent HIV-1 Infection in HIV-Infected Individuals	NCT03212989	Phase 1	University of North Carolina, Chapel Hill	Recruiting

## Prevalence and epidemiology of neuroAIDS

HIV-1 is categorised in group M, N, O, and P depending upon differences in the genome. HIV variations at the molecular level are termed as clades. HIV-1 is further categorized into clade A-K and several recombinant forms (approx. 89). Clade A-D has a high prevalence, compared to others. Clade B is primarily prevalent in developed countries, whereas other clades A, C, E have commonplace in developing or underdeveloped countries. Mutation and evolution of these clades have also been observed [28]. In Southeast Asia and West Africa two recombinant forms viz. CRF01_AE and CRF02_AG are prevalent [29]. Most of the clinical research in America, Western Europe, and Australia has been conducted on clade B, which represents nearly 12% HIV infection globaly. However, very few studies have been performed on C clade, which represents 50% HIV infection globaly. In Africa and India C clade is prevalent more. The existing ART basically meant for HIV-1 clade B [30]. Morover, in Sweden the ART has showen ineffectiveness on HIV-1 clade C [31]. Nearly 31% og untreated HIV-1 clade A and D infacted patients exhibits HAD [32].

The sequence of aminoacids of toxic viral protein (Vp) and Trans-activator of transcription (Tat) governs the level of neurological complications [33]. The cognitive impairment due to HIV-1 clade C infection in South African is found similar to other HIV clades [34]. The prevalence of neuroAIDS showed a linear increase in developed countries, whereas an exponential rise in middle or low income countries. It has been found that nearly 50% of people with dementia are from rich countries, 39% are from middle-income countries, and only 14% are from low-income countries. The primary factor for dementia is the age; a longer life expectancy leads to more people with dementia. A report says that every 20 years, the number of people with dementia doubles and predicted 65.7 million in 2030 and 115.4 million in 2050. This will impact mostly on developing countries [35]. The global death rate due to dementia for the male is ~ 6.7 per 100,000, and for females, 7.7 per 100,000. According to the World Health Organization (WHO), in India, the dementia mortality rate is 13.5 per 100,000 males and 11.1 per 100,000 females [36].

UNAIDS has reported that the COVID-19 pandemic could impact the low- and middle-income countries on supplies of the generic antiretroviral medicines. The lockdowns and border seals as a preventive measure against COVID-19 are affecting the production and distribution of drugs, resulting in an increased cost and supply issues. It is predicted that the final cost of antiretroviral medicines for exportation from India could be 10 - 25% higher than standard prices. It has been estimated that a complete six-month disruption in HIV medication could lead to more than 500,000 additional AIDS-related deaths. If services to prevent mother-to-child transmission of HIV were similarly halted for six months, the estimated increases in new child HIV infections would be 162% in Malawi, 139% in Uganda, 106% in Zimbabwe, and 83% in Mozambique [4].

## Neuropathology of neuroAIDS

In HAND and neuropsychological impairment (NPI), CD4 count is decreased [37]. In the brain autopsy of HIV infected patients, it has been observed that gray and white matter gets damaged. Furthermore, in the white matter where CD20+ B lymphocytes and CD8+ T lymphocytes are prevalent, the perivascular lymphocytic cuffing and low-grade lymphocytic meningitis have been detected [38]. A declined level of gray and white matter has been observed in parietal, frontal, and temporal lobes of a HAD patient. HIV proteins, such as Tat, Viral Protein R (Vpr), and Glycoprotein 120 (Gp120), cause neuron cell death by activating TNF-α, IL-6, and IL-1, intracellular calcium ion load, and high ROS (Reactive oxygen species) generation. HIV encephalitis (HIVE), AIDS dementia complex (ADC), neurodegeneration, necrotizing lesions, neurosyphilis, meningitis, neuropathies, vacuolar myelopathy, leukoencephalopathy, and CNS lymphomas are common disorders in neuroAIDS [14]. In HIVE, multinucleated giant cells (MGCs) and pallor of white matter, neuronal apoptosis in the hippocampus with microglia activation are observed [39]. In the brain tissue extract of HIVE patients, Tat mRNA and protein have been detected [40]. Tat protein has been traced in CSF samples of HIV patients by ELISA [41].

## Mechanism of neuropathogenesis in neuroAIDS

**Fig. 1:**
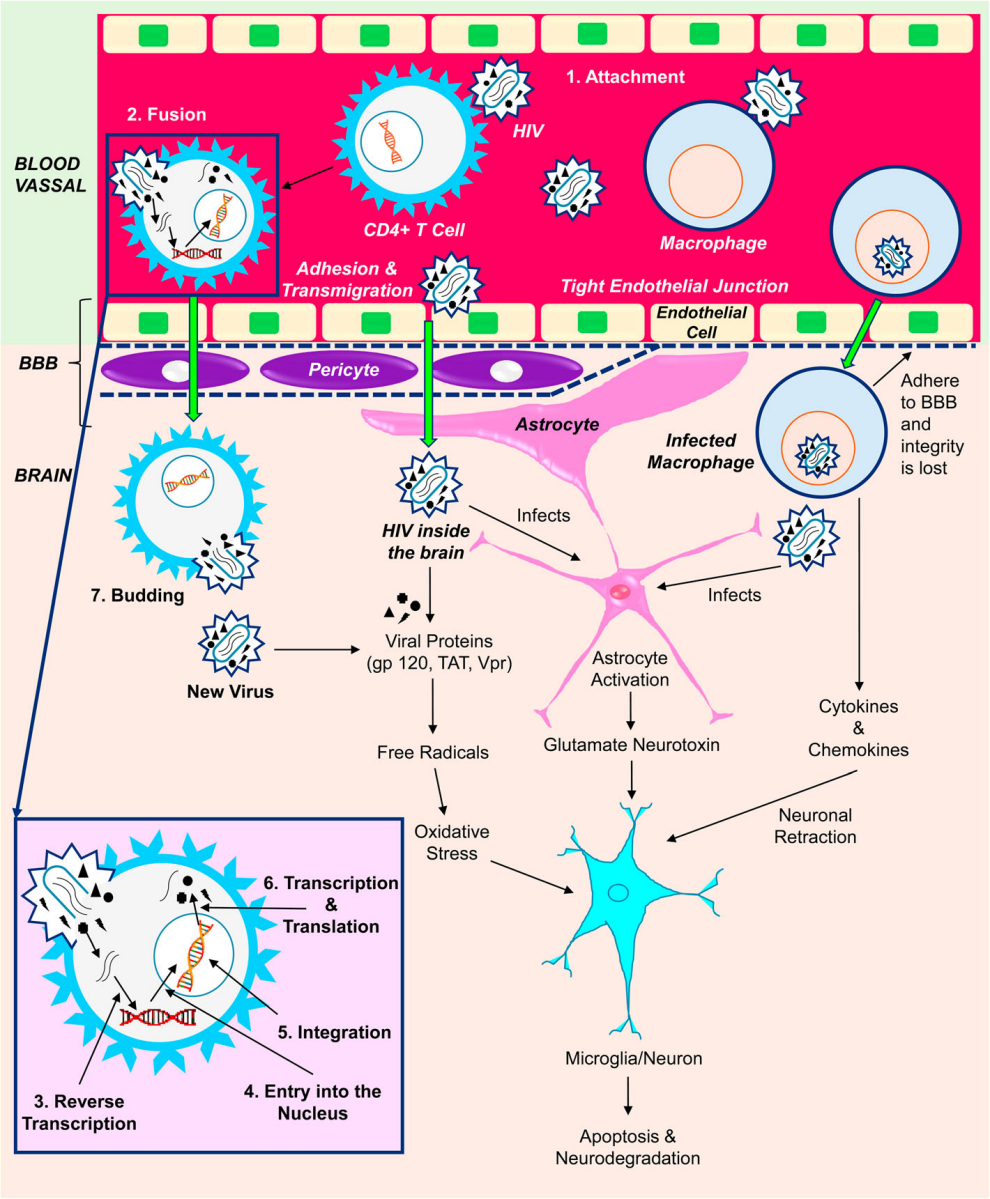
Mechanism of viral infection and neurodegradation in neuroAIDS. The HIV can enter into the brain of the infected people across the BBB, BCSFB at the early stage via CD4, CXCR4, CCR5 receptors bearing cells such as lymphocytes, monocytes, and macrophages. The adhesion of these HIV infected cells to the endothelial layer of BBB, loosen its integrity, and thus facilitate the infected lymphocytes, monocytes, macrophages including free HIV to enter inside the CNS. They replicate in microglia and macrophages of brain, and thus create a HIV reservoir and provoke neuroAIDS. The neurodegradation in neuroAIDS is associated with the production and harmful effects of cytokines (TNF-α, IL-1β), chemokines and viral proteins (gp120, TAT and Vpr) on brain astrocytes and glial cells leading to necrosis/ apoptosis

HIV gets the entry to the host body through the mucosal surface and reached lymph nodes. The main target of viral proteins of HIV are CD4+ T lymphocytes along with chemokine receptors type 5 (CCR5) or type 4 (CXCR4). As a consequence, the fusion of the viral envelop and the cell membrane of lymphocyte takes place (Fig. 1). Other CD4 and chemokine receptors bearing cells such as monocytes, macrophages, and dendritic cells are also the targets of HIV [42–44].

This HIV can enter into the brain of the infected people across the BBB, blood-cerebrospinal fluid barrier at the early stage via Trojan Horse” effect by infecting lymphocytes and monocytes [45, 46]. Few studies have revealed that free virus can also be able to cross the BBB and hence get the entry into CNS and infect macrophage and microglia [47–50].

NeuroAIDS reflects a cluster of neurological complications, as mentioned above, caused primarily due to the damage of CNS and PNS by HIV [51]. The neuropathogenesis (Fig. 1) of neuroAIDS covers the HIV associated neurodegradation, toxic effects of cytokines and viral proteins along with immune reactions [52]. Significant neurodegeneration is observed in the basal ganglion of cerebral hemisphere, brainstem, and white matter of brain. The cytokines produced due to the HIV infection such as TNF-α, IL-1β, and chemokines along with the viral proteins gp120, TAT and Vpr cause harm to the astrocytes and glial cells [7]. The infected macrophages/monocytes, by adhering to the endothelial layer of BBB, loosen the integrity of BBB and thereby facilitate the infected lymphocytes, macrophases and HIV to enter inside the CNS. The gp120 and Tat proteins are neurotoxic in nature, where they cause oxidative stress through free radical generation, with subsequent inflammation that potetiate the pathogenesis of neuroAIDS [53–57]. Apoptosis of normal cells can occure directly through the interaction between gp120 protein and N-Methyl- D-aspartate (NMDA) receptor and indirectly through TNF production from nonneuronal cells. Vpr protein can cause cell cycle arrest of the neuronal cell via the caspase-8-dependent process [55, 58, 59]. The Macrophage-tropic (mactropic) HIV-1 R5 strains can infect low CD4 expressed macrophage, but non-macrophagetropic (non-mac-tropic) HIV-1 R5 strains need highly CD4 expressed CD4+ T Cells [60]. Highly macrophagetropic HIV- 1 R5 strain is mostly found in brain tissue and CSF, whereas its detection is infrequent in immune cells or in the blood, even in the latter stage of the disease [61]. They replicate in microglia and macrophages of brain, and thus create a HIV reservoir. The progression of the disease can be described by the fusion of HIV infected T cells with macrophages, which results in rapid and massive transfer of R5 tropic viruses consequence into further fusion with neighbouring non-infected macrophages [62]. The HIV reservoir of CNS can reinfect the peripheral cells in the later stages of infection [63]. Although cART can reduce the viral count bellow its detectable level but the inadequate availability of ARVs in the CNS represents a safeguard of the virus inside the brain, thus provoke neuroAIDS.

## Genetics behind neuroAIDS

Since the last few decades, scientists are focusing on exploration of the genetic basis of neuropsychiatric consequences of HAND and neuroAIDS in AIDS patients. The novel technologies including genetic microarray expression and proteomic are capable of determining the up- or down regulated genes and protein expression during the pathogenesis of HAND.

Levine et al. utilized the weighted gene co-expression network analysis (WGCNA) method to explore gene networks involved in neuroAIDS. The result showed the involvement of the gene networks associated with neurocognitive impairment (NCI) in HIV patients. Differential expression analysis identified the hub genes highly correlated with NCI, that is responsible for neuropathologic processes in HAND. Moreover down regulation of genes involved in mitochondrial functioning has observed [64]. In another study, Yelamanchili et al. explored the significance of microRNAs (miRNAs) in neurodegradation process of HAND in humans and monkeys. The result showed the increased miR-21 expression in the brain neurons of AIDS patients. The miR-21 induction in neurons was due to stimulation of NMDA receptor for prolong time and consequences into neuronal dysfunction and decreased myocyte enhancer factor 2C (MEF2C) expression [65]. Repunte-Canonigo et al. studied about altered gene expression in HIV-1 transgenic (Tg) rats with neuroAIDS and impaired memory. The gene set enrichment analysis (GSEA) algorithm was utilized to assess and report the results. They detected alterations in gene expression supportive of microgliosis and astrogliosis. Among various genes they detected up regulation of the interferon-stimulated gene 15 (ISG-15) in the course of infection in HIV-1 Tg rats. Other gene such as prostaglandin D2 synthase (Ptgds), which is responsible for the activation of immune system and development of astrogliosis and microgliosis was up regulated. They also detected dysregulation of certain genes like IGF, ErbB, and netrin signalling and the PI3K signal transduction pathway, which are involved in neuronal tropism and neurodegenerative diseases [66]. In a study by Siangphoe and Archer found that 411 genes were differentially expressed in HAND and HIVE patients, out of which 94 genes are responsible for immune system activation, interferon response, or antigen presentation. Among these genes 66 genes were heavily up regulated including PSBM8-AS1, APOL6, CTSB, NET1, PLSCR1 etc. They found that only BTN3A2 was expressed in HAND patient with HIV encephalitis [67].

The inadequate permeation of antiretroviral drugs in the HIV reservoirs is due to the over expression of active efflux transporters (AET) over the lymphocytes, macrophages, and the cells that represent the blood-brain (BBB) and blood-cerebrospinal fluid (BCSFB) barriers [68–74].

Most of the antiretroviral drugs are worried to AET systems belonging to the ATP-binding cassette (ABC) gene family such as P-glycoprotein (P-gp-ABCB or MDR gene family), multidrug resistance–associated proteins (MPRs-ABCC gene family), and breast-cancer-resistance protein (BCRP-ABCG gene family) [70, 75–81].

It is well known that xenobiotic enhances the active efflux transporters expression on brain microvascular endothelial cells. For instance, the concomitant exposure of primary human brain microvascular endothelial cells (HBMVEC) to HIV-1 and saquinavir induces an increased MDR-1-mediated drug efflux [82]. Moreover, the PIs able to induce P-gp expression in brain microvessel endothelial cells belonging to the BBB [83–85]. The induction of P-gp in peripheral organs and brain microvessel endothelial cells appears to be mediated through the activation of the nuclear pregnane X (PXR) and constitutive androstane (CAR) receptors [86–91]. Thus the nuclear receptor activity of ligands can further restrict their ability to enter the brain, being able to increase P-gp expression at the BBB level. Taking these aspects into account, it has been suggested that the targeted suppression of P-gp expression in the HIV-1-infected reservoirs of the body may constitute a new strategy able to inhibit antiretroviral drug efflux from the brain [82].

## Treatment strategy of neuroAIDS

### Traditional approaches and challenges

There are ~38 million HIV infected people living in the world. In 2019, ART has reached to 25.4 million people (67% of 38 million). The UNAIDS reported 90-90-90 (90% of all people living with HIV know their HIV status; 90% of all people with diagnosed HIV infection receive antiretroviral therapy; 90% of all people receiving antiretroviral therapy find viral suppression) attainment in 2019, where 81 % of the HIV infected people knew their status of infection, 82 % of HIV positive individual received ART, and 88 % of it showed viral suppression. Now they are aiming for 100–100–100 by 2030 [4]. WHO recommended several ARV drugs as highly active antiretroviral therapy (HAART) for the management and prevention of AIDS progression. ARV can be categorized into several classes, as mentioned in (Table 1). Generally, the HAART regimen comprises of ARVs from different categories. HAART declines the plasma viral load to an undetectable level and increases the life expectancy by tenfold among AIDS patients, thus reduces the mortality rate of AIDS patients [92, 93]. However, irregularities in treatment by HAART can consequence in a relapse of the disease and make a challenge to complete restriction or elimination of HIV infections.

A decline in morbidity rate has been observed among AIDS patients under the HAART treatment. Still, subsequently, an increase in CNS dysfunctions, including minor cognitive impairments/motor disorders, has also been noticed in such patients. During 1998-2008 at least 25% of AIDS patients under HAART treatment developed neurological syndrome [94]. The inefficiency of current HAART regimens for the treatment of neuroAIDS may be ascribed due to some reasons. Foremost, the inflammatory cascades involved in HIV-associated neuronal disorders are not the target of the current HAART regimen. Secondly, the inability of ARV drugs to cross the blood-brain barriers shrinks their effect on the viral particle in the brain/CNS. This may cause the development of ARV resistant viral strain, as noticed in a few cases of AIDS [95].

Moreover, the short half-life and low bioavailability of ARV drugs due to the first-pass effect may also impact on their entry to the CNS [96, 97]. ARV is mostly plasma protein-bound drugs, which limits their content for the CNS [98]. Besides, side effects and overall cost of HAART may also impact on the efficiency of treatment. As a whole, the main reason for the failure of HAART in neuroAIDS treatment is the complexity of barriers in the brain and CNS [99].

Despite noticeable advancement in cART, it is still a challenging task to cross the BBB, BCSFB and bypass efflux transporters. BBB is a set of complex brain microvasculature, composed of three cellular components, namely, endothelial cells, pericytes, and astrocyte [100, 101]. The brain endothelial cells are connected very tightly at their junctions (50-100 folds higher than the other cell counterparts) with nearly 1500 – 2000 Ωcm^2^ electrical resistance [102, 103].

The choroid plexus possesses the BCSFB composed fenestrateried endothelial cells in ventricular region, but the epithelial cells and tanycytes of the choroid plexus possesses tight junctions that restricts the transmigration of ARV from the blood to brain/CNS [104–107].

The efflux transporters of BBB and BCSFB hinder the drug entry inside the brain [108–110]. The ARV nanomedicines have to tackle this for the effective treatment of neuroAIDS. Several novel strategies have been formulated to deliver ARV drugs in the brain/CNS for the management of neuroAIDS with desired features [111].

### Novel approaches and challenges

The availability of ARV drugs in cerebrospinal fluid at therapeutic concentration depends upon the physiochemical properties of the drugs, such as molecular weight, lipophilicity, drug-protein binding and the affinity towards efflux transporters [112]. It was observed that lipophilic drugs such as protease inhibitors (PI) have a high affinity for drug efflux transporter at BBB, thereby prevent their entry into the brain. However, in combination, the PIs of higher affinity will bind to the transporter and thereby preventing the efflux of the co-administered PIs that facilitate its brain entry [113]. To enhance drug delivery to the brain, various strategies such as non-invasive methods, including drug modification to its lipophilic analogues, pro-drugs, chemical drug delivery, carrier-mediated drug delivery, receptor/vector-mediated drug delivery, and intranasal drug delivery are widely used [102, 114–116]. Alternatively, the invasive methods, such as the BBB disruption by osmotic or biochemical means, or direct intracranial drug delivery by intracerebroventricular, intracerebral, or intrathecal administration after creating reversible openings in the brain, are also recognized [117].

Receptor-based targeted drug delivery to the brain has been evolved, but the large size of cargo creating the challenge. Stimuli triggered approaches such as ultrasound driven, and magnetic field-based BBB opening explained effective drug delivery to the brain [1].

Nanocarriers such as polymeric nanoparticles, liposomes, solid lipid nanoparticles (SLNs), and micelles can facilitate drug transmigration into the brain through endocytosis by inhibiting ABC transporters of the BBB [97]. Nanocarriers, with its small size and increased surface area, offer great potential in therapeutic delivery [118]. Besides, they can be manipulated in terms of size, shape, and surface engineering to favour the drug uptake, release, and ingress across the BBB [107, 119]. For instance, polysorbate 80 coated or apolipoprotein E tagged nanocarriers can guarantee the drug delivery across the BBB [120]. Following transferrin (Tf) conjugated saquinavir and amprenavir loaded nanoparticle can cross BBB via a receptor-mediated transcytosis. Significant uptake of quantum rod QR-Tf-saquinavir or quantum dot QD-Tf-amprenavir by Bovine Brain Microvascular Endothelial Cell (BMVECs) along with a substantial enhancement in transmigration capability of these drugs across BBB, as well as a decline in HIV-1 viral replication in peripheral blood mononuclear cells (PBMCs) are observed [121, 122]. The novel macrophage-carriage system can facilitate PIs (atazanavir, ritonavir, indinavir, and efavirenz) entry into the brain [123–125]. Nanogel carriers are made up of the network of poly (ethylene glycol) (PEG)- or pluronic-polyethyleneimine (PEI), star PEG-PEI or poly (amidoamine) dendrimer-PEIPEG. The nanogels are functionalized with brain-targeting peptide specifically binding to the apolipoprotein E receptor. Brain-targeting peptides decorated nano-NRTIs exhibit increased antiviral efficacy with reduced mitochondrial DNA toxicity [126]. Moreover, CRM197-grafted polybutylcyanoacrylate (PBCA) nanoparticles exhibited enhanced uptake by HBMECs, thereby increase the permeability coefficient of zidovudine across BBB [127]. Jayant et al. developed nanoformulation using a layer-by-layer approach containing tenofovir and vorinostat to explore the shock-and-kills method for HIV eradication [128]. This nanoformulation can cross the BBB under a magnetic field, and released drugs first activated the virus then subsequently suppress viral replication. Magnetically guided strategy allow MENPs-AZTTP nanoformulation to cross the BBB, and further on application of alternating current (ac)-magnetic field drug was released, eradicate the virus in an on-demand manner. Similarly, a magnetic-guided delivery method has also been used to deliver brain-derived neurotrophic factor (BDNF) and TIMP-1 for neuroprotection of HIV-infected brain [129, 130]. Further, magnetically guided delivery of Beclin1siRNA through the BBB was investigated to reduce HIV induced inflammation. Here Beclin1siRNA was coupled with MENPs and further released via ac-magnetic stimulation [131]. Chiappetta et al. formulated efavirenz (EFV)-loaded polymeric micelles for the treatment of HIV/AIDS in children [132]. For the management of neuroAIDS through neuroprotection, various strategies were developed, for example, microphage-driven approach [123], long-acting therapeutic agent [133, 134], novel nano-NRTI formulations [135], and new techniques to cross the BBB [136]. Despite significant advancements in terms of neuroAIDS eradication, the neuroprotection through novel strategies via the invasive route of intravenous administration is a big challenge. Considering the anatomical advantages of the nasal cavity, the intranasal route may be an excellent alternative to deliver antiretroviral drugs directly from the nose to the brain.

## Anatomy and physiology of nasal cavity

**Fig. 2:**
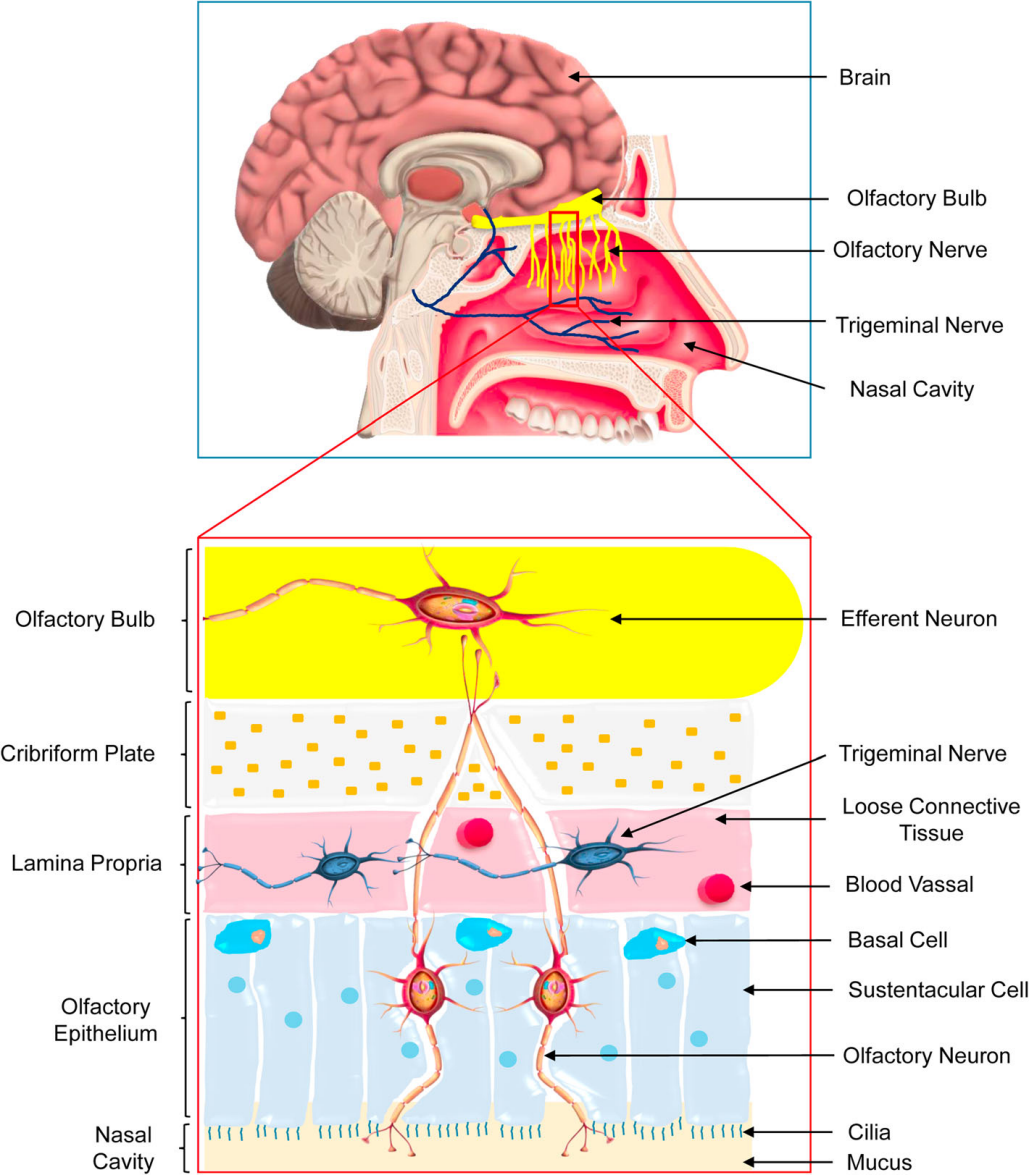
Anatomy of human nasal cavity. The nasal cavity is divided into the vestibule, respiratory and olfactory sections. The nasal vestibule is the dilated area at the nostril opening. The respiratory section of the nasal cavity refers to the passages through which air travels into the respiratory system. The high level of vascularization and the presence of microvilli make the respiratory epithelium as the primary site for systemic drug absorption. The olfactory region of human nasal cavity (2-12.5 cm2) represents only 1.25 - 10% with a thickness of 60 μm. The olfactory epithelium provides an option for the entry of drug/ formulation directly into the brain

For the development of a compelling nose to brain delivery system, it is essential to know the exact mechanism involved in the transport of drugs through nasal rout. Therefore, the proper knowledge of the anatomy and physiology of nasal route is utmost important (Fig. 2).

### The nasal cavity

Beside the mouth, the nose comprising two nostrils which provide an external opening to enter air passing through the nasal cavity upto the lower lung. The nasal cavity performs a vital task like humidification and regulation of inspired air temperature, particle filtration, and olfaction [137–139]. Anatomically the nose is divided into two segments by nasal septum longitudinally [140]. Both the parts have three distinct regions, the vestibule (0.6 cm^2^), the olfactory (2 - 12.5 cm^2^) and the respiratory regions [137, 140–142]. The length of the nasal cavity is 12 - 14 cm with a height of 5 cm, and it covers 150 - 200 cm^2^ surface area with total volume of 13 - 25 ml [137–140, 143, 144]. The vast nasal surface area is composed of superior, middle, and inferior nasal conchae (or turbinates). The turbinates are lined by nasal mucosa that plays a vital role in regulating the warmth, humidity of the inhaled air [140, 142]. The inhaled air passes through the nasal vestibule into the main nasal chamber through the flexible nasal valve [139, 142, 145]. However, the olfactory region is touched by only 15 - 20% of the inhaled air as per the structure of the nasal cavity [145].

### The respiratory epithelium

The respiratory epithelium covers 80 - 90% of the nasal cavity, which is composed of ciliated pseudo stratified columnar epithelium lies over the respiratory region [138, 140, 141]. The high level of vascularization and the presence of microvilli make the respiratory epithelium as the primary site for systemic drug absorption [138, 145, 146]. Blood is perfused to this region through the maxillary artery [146]. The main functions of the respiratory epithelium are to coordinate the ciliary movement, exchange of water and ions between cells, the secretion of mucus, and clearance along with humidity of the mucosa. The respiratory epithelium is composed of the ciliated and non-ciliated columnar cells, basal cells, and goblet cells [137, 141]. The entire respiratory epithelium is surrounded by a pericilliary layer (3 - 5 μm) and the overlying dense gel layer (2 - 4 μm) [145, 147, 148]. This mucus gel is composed of mucins, water, salts, proteins, and lipids. This acts as a defensive barrier against inhaled particulate matters [149, 150].

### The olfactory epithelium

Due to the special ability of the olfactory epithelium to provide an option for the entry of medicines directly to the brain, it has gained importance among the researcher. The olfactory mucosa is composed of a ciliated sensory pseudo stratified columnar epithelium. Anatomically it is surrounded by respiratory epithelium and held on the superior turbinate and bilaterally on the nasal septum. Beneath the olfactory epithelium contains the lamina propria. It also possesses a dense network of blood capillaries from the ophthalmic artery, lymphatic vessels, olfactory axon, autonomic nerves, the trigeminal nerves, and the mucus-secreting Bowman’s glands [141, 144, 146]. The nonmotile cilia (50 μm) are present on the olfactory epithelium [144]. The olfactory region of human nasal cavity (2 - 12.5 cm^2^) represents only 1.25 - 10% with a thickness of 60 μm [140–142, 145]. The morphology of the olfactory system determines the sensing ability and olfaction between humans and other species [138, 142, 151]. The mucus layer cleans up the sensory region and solubilizes odoriferous substances along with foreign entities [141]. The olfactory epithelium contains several cell types, including sustentacular cells, which provide metabolic and mechanical support along with ionic balance, basal cells, brush cells [137, 140–142, 144].

## Mechanism of the nose to brain delivery

**Fig. 3:**
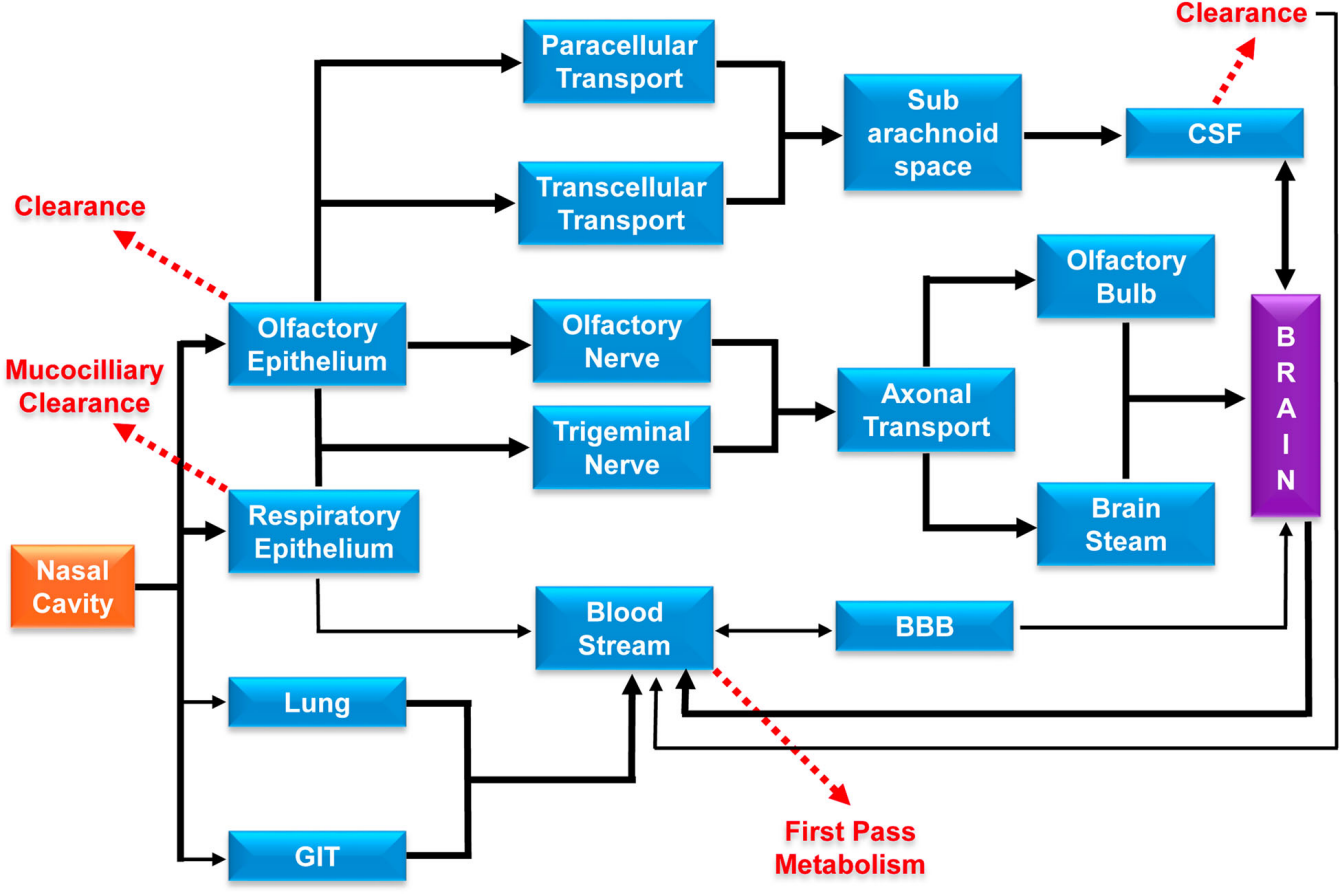
Various nose to brain drug transport pathways. After nasal administration of drug, it can reach the brain via systemic, olfactory and trigeminal pathways based on drug absorption site/ time, physicochemical nature of the drug/ formulation and mode of application. The olfactory and trigeminal pathways can bypass first-pass metabolism of drug and BBB to deliver the drug inside the brain via paracellular/ transcellular routes of drug transport

The direct nose to brain delivery of some therapeutic agents through the intranasal route has been investigated in numerous research studies. It was observed that a small portion of the administered dose can access the brain, which reflects that the exact underlying mechanisms are yet to be explored. Understanding of the actual pathways involved in the nose to brain delivery of drugs would facilitate the production of optimized formulations for CNS diseases. However, several transport pathways have been proposed, such as systemic olfactory and trigeminal nerve pathways (Fig. 3) [145, 152–154]. These pathways differ from each other via the drug absorption site and the absorption time. The superiority of the transport pathway is dependent on the physicochemical character of the drug or the formulation and the mode of application [146].

### The systemic pathway

As the nasal respiratory epithelium is highly vascularised; therefore, there is a chance of indirect nose to brain delivery of drugs/formulation [145, 152]. Before the absorption into the systemic circulation, the elimination processes (enzymatic activity and mucociliary clearance) must be bypassed by the drug in the nasal cavity and subsequently need to cross the BBB to reach the brain [146, 152, 154]. In general, low molecular weight lipophilic substances favour this pathway [151, 155]. Whereas hydrophilic drugs follow paracellular route and exhibit higher bioavailability compared to low molecular weight lipophilic drug [144]. The positive surface charge, structures, and shapes also impact on the permeation across the BBB [156, 157]. Additionally, short half-life, protein binding and altered pharmacokinetic properties of drugs also has a detrimental effect on BBB permeation [152, 157]. Few studies on animals have suggested that drugs can transmit from venous to the carotid artery and subsequently into the brain via a local counter-current mechanism [146, 158–162]. Alternatively, drug transfer from blood to the brain can take place across the choroid plexus [163, 164].

### The olfactory pathway

Drug delivery to Brain/CNS through olfactory mucosa has been widely investigated due to its high exposure in the CNS, CSF and olfactory bulb, rapid absorption, deceased systemic effect, and ability to bypass the BBB [138]. The olfactory pathway involves neuronal, extracellular or intracellular route [138, 146, 153, 165]. Large molecule like protein and peptide delivery has been investigated through this transnasal route. Kang et al. 2010 have investigated the combinatorial neuro protective effect of erythropoietin (EPO) and insulin-like growth factor-I (IGF-I) in neuroAIDS. After transnasal treatment with EPO+IGF-I, in addition to neuroprotection, activation of Akt (protein kinase B) and inhibition of glycogen synthase kinase (GSK)-3β have been observed with decreasing downstream hyperphosphorylation of tau (responsible for neurodegradation) in transgenic mouse brain. These results depict that the peptides enter into the brain post transnasal administration and affected their cognate signaling pathways within the mouse brain [166]. In another study, Thaney et al. 2017 showed the neuroprotective effect of interferons β (IFNβ) in neuroAIDS. They reported the neuroprotection by intranasal IFNβ treatment of transgenic mice expressing HIV-1 envelope glycoprotein 120 in their central nervous system (HIVgp120tg). They observed in *in vitro* cerebrocortical cell cultures that the neuroprotection by IFNβ against gp120 toxicity was dependent on IFNα receptor 1 (IFNAR1) and the β-chemokine CCL4. They estimated the significant amount of *in vivo* IFNβ mRNA in HIVgp120tg mice brains, which is responsible for an increased expression of CCL4 and protection against HIVgp120-induced brain injury [167].

The drugs/formulations are internalized into olfactory neurons through endocytosis transported into the olfactory bulb and thereby enter into the CNS [153, 168, 169]. The diameter of the human olfactory axons is 0.1 - 0.7 μm [170]. Some experiments have been done to check the axonal transport of some therapeutic agents [171–175]. Gottofrey and coworkers investigated the radiolabeled cadmium (109CD2+) transport through the olfactory region [174]. In another study by Thorne and fellow orkers investigated the transport of IGF-I to the rat brain through olfactory axon [175]. In a different study, the wheat germ agglutinin conjugated polyethylene glycol-polylactic acid (PEGPLA) nanoparticles (WGA-NP) exhibited the involvement of the olfactory neuronal pathway for the delivery of the nanoparticles to the brain [176]. These studies showed slow and inefficient drug transport to the brain [138, 146, 155, 176]. However, some reports depict faster transport, which is dependent on some factors such as the transported substance, the species, the axon diameter [171, 173, 174, 177]. The olfactory epithelium is also takes part in the delivery of agents to the brain, such as insulin, nerve growth factor (NGF), dihydroergotamine, lidocaine [178–182]. However, very few reports have been published to show the transport efficiency of nanoparticles (NPs) through this pathway [155, 176, 183]. The epithelial path is faster than axonal transport [146, 155, 184]. Both intracellular and extracellular mechanisms are involved in the transportation of drugs across the olfactory mucosa. The mucosal gel layer permeation is essential for drug transport via transcellular or paracellular pathways [140, 143, 168].

### The trigeminal nerve pathway

The trigeminal nerve is the largest cranial nerve that carries sensory information to the oral, ocular, and nasal mucosa [137, 185, 186]. The trigeminal nerves are innervated in both the respiratory and olfactory mucosa. It offers an alternative route for the nose to brain delivery of drugs/formulations [152, 187, 188]. The branches of the trigeminal nerve from the ophthalmic and maxillary region innervate the dorsal and lateral nasal mucosa along with the anterior part of the nasal cavity [140, 168]. These trigeminal nerve branches synapse at the trigeminal ganglion and enter through pons of the brainstem and subsequently directed to the hindbrain and forebrain [137, 152, 168]. Various studies have been reported on the transport of several agents such as Insulin-like growth factor 1 (IGF-1), lidocaine, Interferon-β-1b (IFNβ-1b), WGA-HRP through the axon of trigeminal nerves post intranasal administration [175, 187, 189, 190].

### The lymphatic pathway

The olfactory region possesses several extracellular pathways at the submucosal level for drug transport. These extracellular pathways include perineural, perivascular, or lymphatic channels, which extended into the olfactory bulb of the brain. Additionally, clearance of a drug can also take place from the olfactory submucosa via olfactory blood or lymphatic vessels into the deep cervical lymph nodes in the neck. Although these pathways are not clear, a report has been published that describes the connection between the subarachnoid space, nasal mucosa, and deep cervical lymph nodes [140, 191].

## Advantages and disadvantages of the nose to brain delivery

BBB is a complex network of tightly packed endothelial cells of blood vessels in the brain that separate it from the circulatory system. It acts as a protective barrier against toxic substances like various chemicals and toxins. Hydrophilic drugs, molecules having surface charge, proteins, and peptides are not permeable across BBB, while lipophilic substances like antidepressant, anxiolytic and hormonal drugs can move quickly across this barrier [192, 193]. Patients with neurological complications require a long term dosage regimen, consequences into adverse effects in non-targeted organs. The majority of neurotropic drugs have lost their potential due to the BBB, leading to fewer therapeutic options available for neurodegenerative diseases [194]. The non-invasive approach is preferable in the case of neurological disorders for drug therapy. Transmucosal drug delivery via olfactory or trigeminal pathways can directly transport drugs to the brain bypassing the BBB. Through this route, only the brain is connected with the outside environment [137]. The disadvantages of this include, it requires small administered volume, possesses less surface area, the short residence time for drug absorption, and the influence of the physiology of the nasal cavity [195].

## Nose to brain delivery determining factors

### Factors affecting the nose to brain transport of drug

The physiological characteristics of a nasal environment determine the effectiveness of nose to brain drug delivery. The various metabolic enzymes, osmolality, and pH (4.5 - 6.5) of the nasal cavity may affect drug metabolism and its effect [196–198]. The physicochemical properties of drug like size, lipophilicity, and degree of ionization also have an impact on the nose to brain drug delivery. Moreover, the pH, tonicity, drug concentration, viscosity, surfactant, and nature of the dosage form also impact on the absorption [193]. For example, the stability of a drug depends upon the formulation pH and its degree of ionization, and it may irritate nasal mucosa. The tonicity of the formulation interferes with the movement of cilia and thus affects drug absorption. Viscous formulations may increase contact time with the nasal mucosa, but it may affect drug diffusion [199]. The drug administration in the supine position is better for the drug to reach the olfactory region. The optimal volume for the intranasal administration in one nostril is 5 μL in the case of mice and 50 μL in the case of rats, whereas 200 μL in the human nasal cavity [200]. The physicochemical properties of drugs are also responsible for selecting the transport pathway. The lipophilic drugs prefer the transcellular route, whereas the extracellular route is utilized by the hydrophilic molecules.

### Formulation factors in nose to brain delivery

The various hindrance of nasal delivery has been sort out by several formulation approaches. The permeation enhancers are used to eliminate the limitations of nasal absorption of drugs [201]. However, their clinical use is limited, as permeation enhancers may show a toxic effect after chronic use [201]. The contact time of the formulation with the mucus layer of the nasal cavity can be enhanced by using mucoadhesive polymers that reduce the fast mucociliary clearance [202]. The enzymatic degradation of the drug can be reduced by encapsulating within a particle, thereby increasing transport [203].

Nano formulations can establish a good interaction with the olfactory region, which helps in endocytosis by the neurons and supporting cells and then drugs released [137, 204] even though the limited access of nanoparticle larger than 100 nm size for axonal entry in the filia olfactoria, the released drug in the nasal mucosa can be transported via paracellular or transcellular pathway through the epithelium [137]. The hydrophilic NPs choose the aqueous paracellular route, while the transcellular route is preferred by hydrophobic ones [205].

#### Particle size

Particle size plays a crucial role in the nanoparticulate nose to brain drug delivery systems. Smaller sized particles quickly pass the mucous layer, whereas the larger particles face resistance to cross the nasal mucosa. It has been reported that 100 nm sized polystyrene particles coated with chitosan (C-PS) or polysorbate 80 (P80-PS) NPs were accessed more in olfactory epithelium as compared to 200 nm sized particles. In contrast, none of them were found in the olfactory bulbs [206]. The translocation of ferric oxide NPs (40 nm and 280 nm) in the case of the nose to brain delivery were found to be size-dependent. Smaller sized particles move more easily from one cerebral compartment to another [207]. However, much more efforts are necessary to determine the impact of particle size using more advanced technologies.

#### Surface charge

The mucosa of the nasal cavity is negatively charged; therefore, the positively charged particles interact with it through electrostatic forces and establish bioadhesion for a long period. Polymers such as chitosan and its derivatives are positively charged; therefore they are widely used to formulate NPs for intranasal application [208–216]. Positively charged chitosan can also be utilized for coating NPs [153, 217]. It had been reported that chitosan-PS NPs exhibited the highest mucoadhesion than P80-modified NPs. The bound NPs mainly reside on the mucosa, while the P80 coated NPs penetrate the epithelial cell layer [153]. Few studies have revealed that chitosan-PS NPs showed a detrimental effect on nasal epithelial cells when applied in a buffer pH 6.0 due to the enhanced positive charges compared to the buffer pH 7.4 [153]. The positive charges on nanoparticle surfaces retard translocation and favour the trigeminal pathway, while the negative charges on nanoparticle surfaces favour the olfactory pathway. The fluorescent imaging of both positively and negatively charged NPs exhibits its transport in the brain with residence time up to 48 h post intranasal administration [218].

#### Surface modifications

Surface engineering of NPs with targeting ligands such as cell-penetrating peptides (CPP) can enhance its translocation from the nose to the brain [219]. In a study on *in vitro* model of olfactory cell monolayers, PLGA NPs, NLCs, and chitosan-coated NLCs exhibit 0.7%, 8%, and 22% permeation, respectively. After surface modification with CPPs, Tat, or penetratin, the Tat-chitosan-NLCs and penetratin-PLGA NPs showed 46% and 7% penetration, respectively [220]. Thus, ligand conjugation has an impact on penetration through the nasal epithelium. Lectins, such as wheat germ agglutinin, are a promising targeting ligand for the nose to brain delivery of NPs [217]. Lactoferrin is also a potential targeting ligand for the nose to brain delivery application [221].

## *In vivo* and *in vitro* models of nose to brain delivery

The nose to brain drug delivery utilizes olfactory and respiratory epithelium to transport formulations via paracellular, transcellular, and neuronal pathways [222, 223]. The various testing models of the nose to brain drug delivery can be utilized to detect and test drug absorption and permeation through the nasal route for pharmacokinetic, toxicity, and electrophysiological studies, and drug transporter interaction evaluation. Various *in vitro*, *in vivo*, and *ex vivo* models are utilized to test the nose to brain drug transport. *In vitro* methods perform permeation and diffusion studies, whereas *in vivo* models perform nasal absorption and pharmacokinetic studies, and *ex vivo* techniques perform nasal perfusion study [224].

### *In vivo* models

Proper knowledge of anatomy of nasal cavity of the selected animal model is essential to perform *in vivo* nasal permeation studies. The animal models include rats, mice, rabbits, dogs, sheep, and monkeys. For preliminary studies, rat and mouse models are helpful for the nose to brain drug delivery, whereas for pharmacokinetic studies rabbit, dog, and sheep models are widely employed.

However, due to the difference in anatomy and physiology of nasal cavity of the animal model and human, the *in vivo* results may not have correlation always [245]. None to direct brain transfer of drugs takes place via the olfactory mucosa through the nerve axon, or outside the nerve bypassing the BBB.

In the case of the direct nose to brain delivery, it should be noted that the nasal absorption must avoid first-pass metabolism and protein binding. The dosage for the olfactory region is generally 0.01–1% of oral dosage. Additionally, the drug should be soluble in a few microliters of vehicles. The clearance from the nasal cavity is also speedy.

The formulation for nasal administration is usually applied with a polyethylene tube attached to a micropipette by inserting into the nostrils at a depth of 3 mm in the case of mice or 5 mm in the case of the rat. The volume for the intranasal administration is usually considered 5 μl in the case of mice and 50 μl in the case of rats [225]. During the nasal administration, the animals should keep in a supine position to enhance the drug to reach the upper part of the nasal cavity. In humans, 10% of the nasal cavity is olfactory region with limited access; whereas in mice and rats, 50% of the nasal cavity is the olfactory region. The olfactory area of monkeys and humans is similar [226].

### In vitro models

The *in vivo* studies explain the nasal drug absorption and permeation, whereas the *in vitro* studies explore the mechanism of drug absorption and transport through transnasal route. For this purpose RPMI 2650 and CaCo-2 cell lines are employed as testing model. These *in vitro* models impart the paracellular transport through nasal epithelia. However, these models unable to explain the effect of nasal mucus, mucins, clearance, anatomical and physiological factors on drug transport. Additionally, the donor area does not fully reflect the required transport from the mucosa to the receiving nerves.

#### RPMI 2650 cell culture model

RPMI 2650 cell line is the human nasal epithelial tissues. This cell culture model is primarily for the study of nasal metabolism and toxicity [227, 228]. Therefore it is not preferable for drug transport study; however, it has been used for drug permeation studies [229]. The contamination of this cell culture is a significant problem, although it is suitable for peptides transport and metabolism studies [230].

This model utilizes the air-liquid interface (ALI) and the liquid-covered culture (LCC) culture conditions [231]. The apical and basolateral sides of the LCC model, are filled with culture medium, and it is marked by the presence of flattened ciliated cells, mucin, and highest second-day TEER value. In case of ALI model also, first the apical and basolateral sides are filled with culture medium, after which the apical side is airated and in every alternate days the medium of basolateral side is replaced. It has high resemblance to the *in vivo* nasal tissue and it is composed of several ciliated cells, stronger mucin gene expression, and having maximum TEER on 5^th^ day which last for ten days. Thus ALI conditions could provide an adequate environment for preclinical cytotoxicity and permeability studies compared to LCC [232].

#### CaCo-2 cell culture model

CaCo-2 cell line is optained from human colon carcinoma and it is utilised as a testing model to evaluate the nasal absorption of formulations after its differentiation to various cell monolayers. The CaCo-2 cell culture model is the most switable to intestinal mucosa for the drug absorption and permeability study [231, 233–235].

#### Reconstructed human nasal mucosa model

This three-dimensional nasal mucosa model is constructed with human nasal fibroblast cell on a collagen matrix which is used as growth support for the epithelial cells. This model shows four to five fold increased paracellular permeation than the epithelial cell model. The main disadvantage the model is its complexity. However, using this model passive nasal permeation can be evaluated [228].

### *Ex vivo* models

To determine the toxicity and transmucosal transport of nasal formulation, *ex vivo* testing is performed on the nasal mucosa optained from experimental or slaughtered animals such as pig, sheep, rat, rabbit, dog, monkey etc. and as well as from human. The *ex vivo* studies depicts drug permeation, metabolism, efflux, and toxicity. In contrast, it has some limitations, including the varying thickness of nasal epithelium among animal species and the absence of interstitial movement under the mucosa [245].

Ussing chamber is the widely used *ex vivo* nasal perfusion model. This model is quite simple and easy to perform maintaining tissue viability. The permeability study can provide a quantitative estimation of passive diffusion, active transport, efflux transport along with the identification of routes of transport [236]. The distinguished efflux pumps of the nasal mucosa are examined with and without blocking agents through these models [237]. Besides, using the Ussing chamber model, the drug transport through the nasal respiratory and olfactory mucosa can be compared [238]. Therefore, these *ex vivo* models are very effective for drug screening in the early stage of drug and formulation development.

## Imaging technology of nose to brain delivery system

The use of imaging technology in preclinical studies can provide a great information about the biological fate of the delivered therapeutic agents and disease progression, thereby increase the efficacy of novel intranasal (IN) therapies for clinical translation. So far, in preclinical studies, MRI, PET, SPECT, gamma scintigraphy, bioluminescence, and fluorescence imaging have been utilized. However, ultrasound imaging has not been explored so far for IN imaging, because of the problems in transmitting and receiving of acoustic waves across osseous structures in the clinical megahertz ranges using diagnostic ultrasound transducers. Nevertheless, by using specialized therapeutic transducers ultrasound waves can be focused to enhance transmission and enable therapeutic benefits for IN drug delivery. A few CT studies have been done mostly to explore nasal anatomy and nasal flow dynamics in humans and animals. Perhaps the combined PET and MR has the highest future potential for accessing nose to brain route of drug delivery as the combination can provide high quantitation and sensitivity of molecular imaging with high resolution. However, a few investigations have been conducted so far using this dual-modality approach, which enables understanding of *in vivo* biological processes at a fundamental level. These imaging modalities and their contribution in intranasal drug delivery from both the preclinical and clinical perspective have been reported elsewhere [239].

## Intranasal formulations for neuroAIDS

Despite several advantages and advancements of the nose to brain targeting approach, a minimal effort has been made to utilize this route for the delivery of antiretroviral drugs into the brain to treat neuroAIDS. Chiappetta et al. 2013 explored efavirenz loaded poly (ethylene oxide)–poly (propylene oxide) block copolymer micelles for the direct nose to brain delivery of the formulation. It had been observed that the drug loading capacity and availability of the formulation was dependant on the size and composition of the micelle. The hydrophilic nature of the formulation can enhance drug payload (20–30 mg/ml), and intermediate hydrophobic nature is recommended for the better nose to brain delivery. The results revealed four-time higher bioavailability of the drug compared to the oral route and five-time in the case of the intravenous route [240]. The nanoemulsion comprises of saquinavir mesylate (SQVM) was developed for intranasal administration using a metered-dose device. The experiment on rats resulted in higher SQVM concentration in the brain than the intravenous administration of the drug suspension. No significant adverse effect on sheep nasal mucosa was observed, which depicts its ability to deliver ARVs into the CNS for neuroAIDS management [24]. Didanosine (ddI) loaded chitosan NPs were formulated and administered via intravenous as well as the intranasal route to evaluate the potential of chitosan as a targeted drug carrier. The ratios of concentration in brain/plasma, olfactory bulb/plasma, and CSF/plasma were found significantly higher post nasal administration of chitosan nanoparticles/solution than post intravenous application of didanosine aqueous solution. The result signifies the ability of the chitosan nanocarrier as a delivery system for ARVs into the rat brain at a significant level [241]. Barbi et al. 2015 prepared zidovudine loaded chitosan nanoparticle in sodium tripolyphosphate gel. It was observed that nanometer-sized particles allow greater interaction with the nasal mucosa and permeate its uptake through pig nasal mucosa compared to free AZT [21]. Dalpiaz et al. 2019 developed a prodrug of AZT (U-AZT) by nanoprecipitation method and coated by bile acid salt like taurocholate and ursodeoxycholate. More uptakes were observed in the case of taurocholate coated particles by murine macrophages *in vitro* than that of ursodeoxycholate-coated particles. The *in vivo* study showed the same effect as the subarachnoid spaces containing macrophages is a major unreachable site of HIV sanctuaries in the body. It was observed that the formulation with chitosan exhibit greater uptake of U-AZT in CSF [22]. Being a lipophilic drug efavirenz poorly solubile in water. Belgamwar et al., 2017 has developed a chitosan-g-HPbCD NPs loaded with efavirenz using ionic gelation process for its delivery into the CNS. The nanoformulation was administered through IN route and the targeting index exhibited 12.4 fold increased accumulation in the brain as compared to intravenous solution of efavirenz [242]. Despite the feasibility of use, a very few investigations were conducted on SLNs as drug carrier to the brain for ART. NRTI such as efavirenz were loaded in SLNs for brain delivery through nasal route. The result showed 150 fold increased accumulation of formulation in the brain when compared to oral capsule of efavirenz [243]. Pokharkar et al. 2017 investigated the ability of NLCs for ARV delivery to the brain for effective treatment of neuroAIDS. The melt emulsification ultrasonication method was used to prepare NLCs. The optimized formulations exhibited spherical morphology, high encapsulation efficiency, and long term stability. Upon intranasal administration of the formulation, a significant amount of the drug reached the CSF bypassing the BBB. The result of the study depicts that efficient brain targeting can be achieved by the strategic use of drug carrier and the excipients through nasal route [23]. Pokharkar et al 2015 developed a tenofovir disoproxil fumarate (TDF) loaded hybrid nanocarrier composed of acrylate copolymer (Pemulen TR- 1) and lauric acid with size between 215±2.19 to 736±4.55 nm. The optimized formulation possesses shear thinning property and exhibited anomalous type of drug release. The gel significantly enhances the permeability of TDF through sheep nasal mucosa. Subsequent histopathological investigation ensured the safety of the designed carrier for nasal delivery of TDF. Based on the understanding of physicochemical, molecular, microstructural and stability aspects, the designed hybrid nanocarriers possess the potential to entrap TDF, and accentuate its transnasal flux, thus could be used as a carrier for an effective nasal delivery of TDF [20].

The Drug Delivery Research Laboratory at the Department of Pharmaceutical Sciences Dibrugarh University has also developed effective nose to brain delivery system of TDF loaded NLCs for the treatment of neuroAIDS. The NLCs were prepared using Compretol 888 ATO as solid lipid and oleic acid as liquid lipid. The NLCs were optimized using central composite design. The average particle size of the spherical shaped NLCs was found to be at 94.7±15.70 nm with PDI of 0.380±0.024 and zeta potential was observed at 17.0±3.87 mV along with %EE of 35.5±1.04 %. The cytotoxicity study on bEnd.3 cell line and histopathology study on pig nasal mucosa revealed the safety of the formulation for intranasal use. The *in vivo* pharmacokinetics profile in rat brain showed higher MRT, *C*_max_, and AUC, which implies the effective and sustained delivery of TDF over 24 h from the NLCs. The confocal and fluorescence images of brain cryosections labelled with Caumarin-6 NLCs confirmed the localization and accumulation of NLCs in the brain [244].

## Conclusion and prospects

The induction of cART improved and increase the life expectancy of AIDS patients. Although, HIV induced neuronal abnormalities are common now a days that affect the lifestyle of AIDS patient. The significant studies on the pathogenesis of neuroAIDS indicates several potential drug targets, but BBB is the main challenge for the development of new therapy with ARVs. Therefore, CNS remain act as a viral reservoir site. Currently, great effort has been given to develop new strategies for eradication of HIV from the body by the use of nanotechnology. The prevailed therapies for AIDS mostly covers the peripheral tissues only. Hence, a CNS targeted DDS is of outmost important to develop effective delivery of ARVs in the CNS through the BBB for successful treatment and eradication of AIDS including neuroAIDS. The intranasal route may be a potential strategy to deliver ARVs directly from the nosal cavity to the brain by eliminating the hindrance provided by the BBB. Intranasal riute is attractive as it is non-invasive and can bypass the BBB to target CNS, thereby reduces the systemic side effects. The ability to deliver various drug molecules, proteins, peptides, hormones, stem cells etc through the nasal route exhibit the new insights for the prevention and management of different neuronal diseases. However, a very few investigations are performed in the delivery of antiretroviral drugs via the intranasal route. There is not a very clear view till now, whether the available drug in the brain is transpoted from the nasal cavity after its release from the carrier system or the whole drug carrier system is translocated into the CNS through olfactory and trigeminal nerve pathways from the nasal cavity. Thus, more emphasis has to put on research to determine the exact transport mechanism of nanocarriers to the brain and their biological fate. Again, the delivery of surface engineered carrier systems through passive or active targeting approach would be desirable for further progress in this field.

## Data Availability

Not Applicable

## Abbreviations

ABC: ATP-binding cassette
ADC: AIDS dementia complex
ANI: Asymptomatic neurocognitive impairment
ART: Antiretroviral therapy
ARVs: Antiretrovirals
BCS: Biopharmaceutical Classification System
BCSFB: Blood Cerebrospinal fluid barrier
BDNF: Brain-derived neurotrophic factor
BMVECs: Bovine Brain Microvascular Endothelial Cell
cART: Combined antiretroviral therapy
CCR5: C-C chemokine receptor type 5
CD: Cluster of differentiation
CNS: Central nervous system
CPP: Cell-penetrating peptides
CSF: Cerebrospinal fluid
CT: Computerized tomography
CXCR4: C-X-C chemokine receptor type 4
DNA: Deoxyribonucleic acid
ELISA: Enzyme-linked immunosorbent assay
HAART: Highly active antiretroviral therapy
HAD: HIV-associated dementia
HAND: HIV associated Neurocognitive Disorder
HIV: Human Immunodeficiency Virus
HIVE: HIV encephalitis
MGCs: multinucleated giant cells
MND: Minor neurocognitive disorder
MRI: Magnetic resonance imaging
neuroAID: Neuro Acquired Immunodeficiency Syndrome
NGF: Nerve growth factor
NLCs: Nanostructured Lipid Carriers
NMDA: N-Methyl- D-aspartate
NPI: Neuropsychological impairment
PBCA: Polybutylcyanoacrylate
PBMCs: Peripheral blood mononuclear cells
PEG: Poly (ethylene glycol)
PEI: Pluronic-polyethyleneimine
PET: Positron emission tomography
PNS: Peripheral nervous system
RNA: Ribonucleic acid
ROS: Reactive oxygen species
SLNs: Solid lipid nanoparticles
SPECT: Single-photon emission computed tomography
SQVM: Saquinavir mesylate
TDF: Tenofovir disoproxil fumarate
TEER: Transendothelial electrical resistance
TIMP: Tissue inhibitor of metalloproteinases
US FDA: United States Food and Drug Administration
WGA-HRP: Wheat germ agglutinin coupled with horseradish peroxidase
WHO: World Health Organization
